# Impact of Disease Severity and Age on Outcomes After Laparoscopic Versus Open Appendectomy: A Single-Center Retrospective Cohort Study

**DOI:** 10.3390/life16050706

**Published:** 2026-04-22

**Authors:** Nicolae-Dragoș Mărgăritescu, Stelian-Stefaniță Mogoantă, Tiberiu Stefăniță Țenea Cojan, Laurențiu Augustus Barbu, Liviu Vasile

**Affiliations:** 1 Department of Surgery, Emergency County Hospital, University of Medicine and Pharmacy of Craiova, 2 Petru Rares Street, 200349 Craiova, Romania; dmargaritescu@yahoo.com (N.-D.M.); ssmogo@yahoo.com (S.-S.M.); vliviu777@yahoo.com (L.V.); 2 Department of Surgery, Railway Clinical Hospital Craiova, University of Medicine and Pharmacy of Craiova, 2 Petru Rares Street, 200349 Craiova, Romania; tiberiu.tenea@umfcv.ro

**Keywords:** acute appendicitis, appendectomy, laparoscopic appendectomy, open appendectomy, disease severity, complicated appendicitis, age, length of hospital stay, postoperative outcomes

## Abstract

Background: Acute appendicitis is one of the most common surgical emergencies worldwide. This study aimed to evaluate the impact of disease severity and age on outcomes after laparoscopic versus open appendectomy and to identify predictors of prolonged hospitalization. Methods: A single-center retrospective cohort study was conducted at a tertiary emergency hospital, including 311 adult patients who underwent appendectomy between January 2020 and December 2024. The primary outcome was length of hospital stay (LOS), with prolonged hospitalization defined as LOS > 7 days. Results: The study included 311 patients (mean age 43.3 ± 18.0 years; 58.5% male), of whom 38.6% had complicated appendicitis. Complicated appendicitis was associated with older age and longer hospitalization. The laparoscopic approach was associated with a significantly shorter LOS compared with the open approach (4.43 ± 2.62 vs. 5.68 ± 3.11 days, *p* < 0.001). Increasing age independently predicted complicated appendicitis and prolonged hospitalization, while laparoscopy remained independently associated with reduced LOS after adjustment for measured confounders. No significant interaction was observed between surgical approach and disease severity. Conclusions: Disease severity and age are the main determinants of postoperative outcomes in acute appendicitis. Laparoscopic appendectomy was independently associated with shorter hospital stay after adjustment, although this association may be attenuated in more severe disease.

## 1. Introduction

Acute appendicitis represents one of the most common causes of abdominal pain and remains a major surgical emergency worldwide [1]. The lifetime risk is estimated at approximately 6–8%, and although the disease predominantly affects younger individuals, a second incidence peak has been described in older patients, who frequently present with atypical symptoms and a higher risk of complications [2,3,4].

Appendectomy remains the standard treatment for acute appendicitis, with both open and laparoscopic approaches widely used in clinical practice. Over the past decades, laparoscopic appendectomy has gained increasing acceptance due to its minimally invasive nature and potential advantages, including reduced postoperative pain and shorter hospital stay. However, its superiority over the open approach remains controversial, as several studies have reported comparable outcomes between the two techniques [5,6,7,8,9].

Disease severity plays a central role in determining clinical outcomes. Complicated appendicitis, including abscess formation and generalized peritonitis, is associated with increased morbidity, prolonged hospitalization, and higher healthcare burden. In addition, discrepancies between intraoperative findings and histopathological results have been reported, highlighting challenges in accurately defining disease severity [10,11,12].

Patient-related factors, particularly age, further influence disease presentation and postoperative outcomes. Older patients are more likely to develop complicated forms of appendicitis, experience delayed diagnosis, and require longer hospitalization. Similar to other acute inflammatory conditions, both disease severity and patient characteristics appear to be major determinants of length of hospital stay [13,14].

Although previous studies have compared laparoscopic and open appendectomy, important uncertainties remain regarding the extent to which postoperative outcomes are influenced by surgical approach itself versus disease severity and patient age. In particular, few studies have evaluated the independent and potentially interacting contributions of these factors to length of hospital stay, especially whether the association between surgical approach and outcomes differs according to disease severity. Therefore, the present study aimed to assess the impact of disease severity and age on outcomes following laparoscopic versus open appendectomy and to identify predictors of prolonged hospitalization.

## 2. Materials and Methods

### 2.1. Study Design and Population

**Figure 1:**
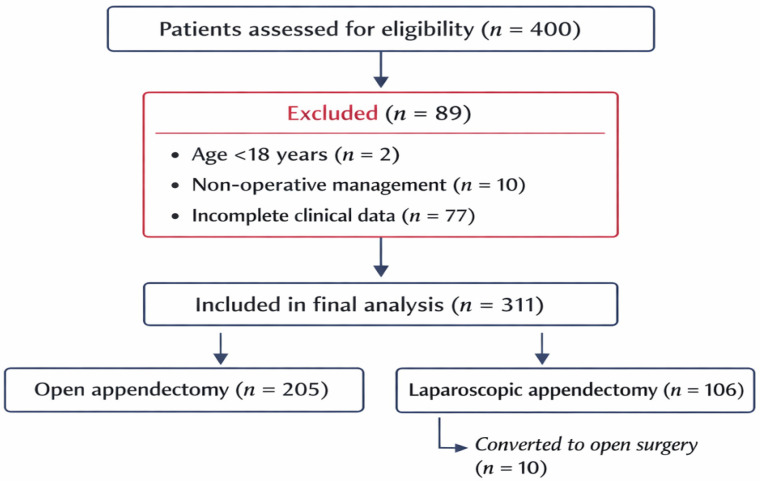
Flowchart showing patient screening, exclusions, final study inclusion, allocation by surgical approach, and conversion from laparoscopy to open surgery.

Patients diagnosed with acute appendicitis were screened for eligibility at the Emergency County Clinical Hospital Craiova, Romania, in the Departments of Surgery I, II, and III, between January 2020 and December 2024. After exclusion of ineligible patients, including those managed non-operatively or with incomplete clinical data, 311 adult patients who underwent appendectomy were included in the final analysis (Figure 1).

Inclusion criteria were patients aged ≥18 years with a confirmed diagnosis of acute appendicitis who underwent either laparoscopic or open appendectomy. Inclusion criteria were patients aged ≥18 years with a confirmed diagnosis of acute appendicitis who underwent either laparoscopic or open appendectomy.

The choice of surgical approach (laparoscopic versus open appendectomy) was made at the discretion of the attending surgeon based on routine clinical practice, taking into account preoperative clinical presentation, suspected disease severity, hemodynamic stability, prior abdominal surgery when known, and surgeon judgment and expertise. Resource availability and institutional practice patterns may also have influenced approach selection. Because treatment allocation was non-randomized, the potential for selection bias and confounding by indication must be considered when interpreting comparisons between groups.

This study was conducted in accordance with the STROBE reporting guidelines for observational studies.

### 2.2. Data Collection

Clinical, demographic, and operative data were retrospectively extracted from electronic medical records. The following variables were recorded: age, sex, type of appendicitis (complicated vs. uncomplicated), surgical approach (laparoscopic vs. open), conversion to open surgery, and length of hospital stay (LOS).

Disease severity was classified primarily according to intraoperative findings documented in the operative reports. For the purposes of this study, complicated appendicitis was defined as appendicitis associated with abscess formation or generalized peritonitis. This definition was selected because these features were consistently documented in the medical records and could be reliably extracted in a retrospective manner. Other pathological descriptors used in broader classifications, such as gangrene, phlegmon, appendiceal mass, or isolated perforation without documented abscess or generalized peritonitis, were not consistently recorded across all cases and were therefore not included in the study definition.

Data on comorbidities, ASA class, BMI, inflammatory markers, and timing-related variables were not consistently available in the medical records and were therefore not included.

### 2.3. Outcomes

The primary outcome was length of hospital stay (LOS). LOS was selected because it was consistently available for all patients and represented a pragmatic and objective measure of postoperative recovery in this retrospective dataset, although it may also be influenced by institutional and nonclinical factors. Prolonged hospitalization was defined as LOS > 7 days.

Secondary outcomes included the association between age, disease severity, and surgical approach with postoperative outcomes, as well as identification of independent predictors of prolonged hospitalization. Additional postoperative outcomes such as complications, readmission, reoperation, surgical-site infection, intra-abdominal abscess, ICU use, and mortality were not consistently available and therefore could not be assessed.

### 2.4. Statistical Analysis

Continuous variables were expressed as mean ± standard deviation (SD) and compared using Student’s *t*-test or Mann–Whitney U test, as appropriate. Categorical variables were presented as counts and percentages and compared using the chi-square test.

Logistic regression analysis was performed to identify predictors of complicated appendicitis and prolonged hospital stay. Multivariable linear regression was used to evaluate independent predictors of length of hospital stay (LOS). Multivariable models were limited to variables available with sufficient completeness in the retrospective dataset, specifically age, sex, appendicitis severity, and surgical approach. Several potentially relevant confounders, including comorbidities, ASA class, BMI, inflammatory markers, timing-related variables, prior abdominal surgery, surgeon experience, and calendar-year effects, were unavailable or incompletely captured and therefore could not be included in the adjusted analyses.

Variables included in multivariable models were selected a priori based on clinical relevance and data availability, rather than by automated univariable screening procedures. Model assumptions were evaluated as appropriate for each model. For linear regression, residual normality and homoscedasticity were assessed through residual diagnostics. Linearity of age was assessed, and no major deviation from linearity was identified. Multicollinearity was assessed using variance inflation factors, with no evidence of problematic collinearity. Because patients with incomplete clinical data were excluded, analyses were conducted using complete-case analysis.

Receiver operating characteristic (ROC) analysis was conducted to assess the predictive performance of age for complicated appendicitis, and the optimal cut-off value was determined using the Youden index.

Interaction analysis was performed to evaluate the effect of surgical approach across different levels of disease severity.

A *p*-value < 0.05 was considered statistically significant. Statistical analyses were performed using IBM SPSS Statistics version 26.0 (IBM Corp., Armonk, NY, USA).

No a priori sample size calculation was performed due to the retrospective design of the study.

### 2.5. Ethical Considerations

The study was conducted in accordance with the Declaration of Helsinki and was approved by the Ethics Committee of the Emergency County Clinical Hospital Craiova (approval no. 14827, 20 March 2026). Due to the retrospective design, the requirement for informed consent was waived.

## 3. Results

Table 1 presents the baseline characteristics of the study population (n = 311). The mean age was 43.28 ± 17.97 years (range 18–86), with a predominance of male patients (58.5%). Uncomplicated appendicitis was more frequent (61.4%) than complicated appendicitis (38.6%). The mean length of hospital stay was 5.09 ± 2.94 days.

Uncomplicated appendicitis was the most frequent clinical form (n = 149), followed by appendicitis with abscess (n = 107) and appendicitis with generalized peritonitis (n = 55) (Table 2). Patients with generalized peritonitis had the highest mean age and the longest hospital stay. Prolonged hospitalization (LOS > 7 days) was more frequent in patients with more severe forms of appendicitis.

Baseline characteristics according to surgical approach are shown in Table 3. Patients undergoing open appendectomy were older and more likely to present with complicated appendicitis than those undergoing laparoscopic appendectomy (both *p* ≤ 0.003), suggesting potential case selection. There was no significant difference in sex distribution between groups. The laparoscopic group showed a significantly shorter length of hospital stay compared to the open group (4.43 ± 2.62 vs. 5.68 ± 3.11 days, *p* < 0.001). Conversion to open surgery occurred in 9.4% of laparoscopic cases.

Age was a significant predictor of complicated appendicitis (OR 1.38, 95% CI 1.20–1.58, *p* = 2.28 × 10^−6^), whereas male sex was not significantly associated with the outcome (OR 1.18, 95% CI 0.73–1.91, *p* = 0.506). The model showed moderate discriminative ability (AUC = 0.669) (Table 4).

Age (OR 1.63, 95% CI 1.29–2.05, *p* = 3.37 × 10^−5^) and complicated appendicitis (OR 3.88, 95% CI 1.67–9.01, *p* = 0.00169) were independent predictors of prolonged hospital stay (>7 days), whereas male sex was not significantly associated with the outcome (OR 0.71, 95% CI 0.32–1.58, *p* = 0.395). The model showed good discriminative ability (AUC = 0.803) (Table 5).

Patients with complicated appendicitis were older and had a significantly longer hospital stay compared to those with uncomplicated appendicitis (*p* < 0.001) (Table 6).

Age and length of hospital stay were significantly higher in patients with complicated appendicitis (*p* < 0.001), while sex was not significantly associated with disease severity (Table 7).

A multivariable linear regression model was constructed to evaluate independent predictors of length of hospital stay (LOS) (Table 8). Increasing age and the presence of complicated appendicitis were independently associated with longer hospitalization. Laparoscopic approach remained independently associated with shorter LOS after adjustment for measured confounders available in the dataset, while sex had no significant effect.

Receiver operating characteristic (ROC) analysis was performed to evaluate the discriminative ability of age for predicting complicated appendicitis (Table 9). The optimal cut-off value determined by the Youden index was 47 years, providing moderate discrimination. The ROC curve demonstrated moderate predictive performance.

An interaction analysis between surgical approach and disease severity was performed to explore whether the benefit of laparoscopy differed according to appendicitis severity (Table 10). The interaction term between surgical approach and disease severity was not statistically significant (*p* = 0.41), and no evidence of effect modification was detected in this sample. However, given the limited sample size, the study may have been underpowered to detect interaction effects.

Subgroup analysis according to age demonstrated significantly longer hospitalization in patients aged ≥60 years compared with younger patients (Table 11).

Sensitivity analysis excluding extreme LOS values (>14 days) showed similar results, confirming the robustness of the primary findings.

## 4. Discussion

The present study highlights the combined impact of disease severity and patient age on postoperative outcomes following appendectomy, while also supporting an independent association of the laparoscopic approach with shorter hospital stay. These findings support the growing body of evidence suggesting that both host-related factors and disease-specific characteristics are central determinants of clinical evolution in acute appendicitis.

In our cohort, complicated appendicitis was strongly associated with older age and prolonged hospital stay. This observation is consistent with previous studies demonstrating that elderly patients are more likely to present with advanced disease, often due to atypical clinical manifestations and delayed diagnosis [15,16,17]. Age-related changes in immune response, reduced physiological reserve, and a higher burden of comorbidities may contribute to more severe inflammatory processes and impaired recovery [18,19,20]. Furthermore, elderly patients frequently exhibit blunted inflammatory responses, which can obscure early diagnosis and facilitate disease progression [10,21,22,23]. These findings reinforce the concept that age is not merely a demographic variable but reflects underlying biological vulnerability that significantly influences disease severity and postoperative outcomes.

Acute appendicitis in immunocompromised patients represents a rare but clinically challenging condition. In these patients, atypical presentation, blunted inflammatory response, and delayed diagnosis may increase the risk of perforation, severe infection, and adverse postoperative outcomes. Immunodeficiency-related comorbidities may also complicate perioperative management and recovery. Although immunodeficiency was not assessed in our cohort, these considerations further support the importance of individualized risk assessment in vulnerable patient populations.

Diagnostic uncertainty may also contribute to worse outcomes, particularly in older patients with atypical presentations. Delayed or challenging diagnosis may influence disease severity at presentation and thereby affect postoperative recovery and length of hospital stay [24,25,26,27,28]. This consideration further supports the interaction between patient-related factors and disease severity as determinants of clinical outcomes.

Incidental appendiceal neoplasms represent an additional consideration in patients undergoing emergency appendectomy, particularly in older patients or those with atypical presentations. Although appendiceal tumors were not specifically evaluated in our cohort, their potential occurrence supports the importance of routine histopathological assessment when clinically indicated.

The COVID-19 pandemic may also have influenced patterns of appendicitis presentation, with prior studies reporting delayed presentation and increased disease severity in some settings. Although pandemic-related temporal effects were not specifically analyzed in our cohort, this may represent an additional factor influencing presentation patterns during the study period.

The progressive increase in length of hospital stay across the spectrum of disease severity observed in our study further supports the hypothesis that complicated and uncomplicated appendicitis may represent distinct clinical entities rather than sequential stages of the same pathological process. Recent literature suggests that these forms differ not only in clinical presentation but also in their underlying immunological and microbiological profiles [10,29]. For example, perforated appendicitis has been associated with a more aggressive inflammatory cascade and altered host response compared to uncomplicated disease. Our findings, showing clear differences in both age and length of stay between these groups, provide additional clinical support for this evolving paradigm.

Our results also confirm that laparoscopic appendectomy is associated with shorter hospital stay, supporting its role as the preferred surgical approach when feasible. The advantages of laparoscopy, including reduced surgical trauma, decreased postoperative pain, and earlier mobilization, likely contribute to faster recovery and shorter hospitalization. However, the superiority of laparoscopy in complicated appendicitis remains a subject of ongoing debate. While minimally invasive surgery offers clear benefits in uncomplicated cases, its advantage may be attenuated in the presence of extensive inflammation, abscess formation, or peritonitis. Some studies have reported comparable rates of postoperative complications between laparoscopic and open approaches in complicated appendicitis, particularly regarding intra-abdominal abscess formation [30,31,32,33]. However, this remains a debated issue, as some studies have suggested a higher abscess risk after laparoscopic surgery in advanced disease, whereas others have reported comparable rates between approaches. In uncomplicated appendicitis, this risk appears generally low regardless of technique. Although intra-abdominal abscess was not assessed in our dataset, this remains an important consideration when interpreting potential trade-offs between surgical approaches, particularly in severe disease.

An important finding of our study is the independent role of age as a predictor of both complicated appendicitis and prolonged hospitalization. Although the predictive model demonstrated only moderate discriminative ability, this result highlights the clinical relevance of age as a simple and readily available risk factor. Nevertheless, the moderate AUC indicates that age alone is insufficient for accurate risk stratification. Additional variables, such as inflammatory biomarkers, radiological findings, and comorbidity indices, are likely necessary to improve predictive performance. Accurate diagnosis of acute appendicitis relies on the integration of clinical assessment, laboratory markers, and imaging findings. These factors contribute to confirming diagnosis, assessing disease severity, and supporting appropriate management decisions. Indeed, biomarkers such as C-reactive protein and the neutrophil-to-lymphocyte ratio have been identified as predictors of disease severity and postoperative complications [34,35]. Incorporating such parameters into predictive models may further enhance their clinical utility.

Moreover, the relationship between age and postoperative outcomes appears to be nonlinear. Large-scale analyses have demonstrated a progressive increase in morbidity with advancing age, rather than a simple threshold effect [36,37,38,39]. This finding has important methodological implications, suggesting that age should be treated as a continuous variable in statistical models to avoid loss of information and improve predictive accuracy.

In addition to age and disease severity, comorbid conditions play a crucial role in shaping postoperative outcomes. Patients with significant comorbidities, such as chronic liver disease, have been shown to experience higher mortality, longer hospital stay, and increased readmission rates following appendectomy. These findings highlight the importance of comprehensive patient assessment and risk stratification beyond the acute surgical condition [40,41].

Another important aspect is the impact of intraoperative factors, particularly conversion from laparoscopic to open surgery. Although conversion rates are relatively low, conversion has been associated with significantly worse outcomes, including increased morbidity and prolonged hospitalization. Factors such as complicated appendicitis, diffuse peritonitis, and patient comorbidities have been identified as major predictors of conversion. These observations further support the concept that surgical outcomes are primarily driven by disease severity and patient-related factors rather than the surgical technique itself [42,43].

In contrast, sex was not significantly associated with disease severity or length of hospital stay in our study. This finding is consistent with previous literature suggesting that gender does not play a major role in determining clinical outcomes in acute appendicitis [3,44]. Instead, disease-specific and patient-related factors appear to be the dominant determinants.

The role of postoperative complications also warrants consideration. Complicated appendicitis is associated with a higher risk of adverse outcomes, including intra-abdominal abscess formation, wound infections, and prolonged recovery. Large cohort studies have consistently demonstrated increased complication rates and longer hospital stay in patients with complicated disease [45,46]. These findings are in line with our results and further emphasize the clinical burden associated with advanced appendicitis.

Elderly patients represent a particularly high-risk group, with significantly increased rates of complications and prolonged hospitalization. Studies have reported complication rates exceeding 30% in elderly patients with complicated appendicitis [47]. These findings underscore the need for early diagnosis, prompt surgical intervention, and individualized perioperative management in this population.

Furthermore, the overall clinical condition at presentation plays a critical role in determining outcomes. Patients presenting with systemic involvement, such as sepsis or organ dysfunction, have significantly higher mortality rates and longer hospital stay [48,49]. These findings highlight the importance of early recognition and aggressive management of high-risk patients.

From a clinical perspective, our findings have several important implications. First, early identification of patients at risk of complicated appendicitis may allow for more timely intervention and improved outcomes. Second, risk stratification models incorporating age, disease severity, and additional clinical parameters could help guide decision-making and optimize resource allocation. Finally, while laparoscopy remains the preferred approach, surgical strategy should be individualized based on patient characteristics and intraoperative findings.

Taken together, our results suggest that postoperative outcomes in acute appendicitis are primarily driven by disease severity and patient-related factors, while the surgical approach plays a secondary, albeit important, role. These findings support a patient-centered approach to management, emphasizing early diagnosis, appropriate risk stratification, and individualized surgical decision-making.

### Strengths and Limitations

This study has several strengths. First, it includes a relatively large, real-world cohort of patients with acute appendicitis, reflecting routine clinical practice across multiple surgical departments. Second, the study provides a comprehensive evaluation of both disease-related and patient-related factors, allowing for a more nuanced understanding of postoperative outcomes. Third, the use of multivariable regression models and interaction analysis strengthens the robustness of the findings by accounting for measured confounders available in the dataset and exploring the relationship between surgical approach and disease severity. Additionally, the consistency of results across sensitivity analyses supports the reliability of the conclusions.

Differences in baseline characteristics between surgical groups may have contributed to residual confounding despite adjusted analyses and should be considered when interpreting the findings.

However, several limitations should be acknowledged. First, the retrospective design introduces the potential for selection bias and unmeasured confounding. Second, multivariable adjustment was limited to variables available in the dataset, and several potentially important confounders—including comorbidities, ASA class, BMI, inflammatory markers, timing-related variables, prior abdominal surgery, surgeon experience, and calendar-year effects—were unavailable or incompletely captured and therefore could not be included in the models. Additionally, operative duration was not consistently available and therefore could not be evaluated as a potential predictor of postoperative outcomes. As a result, residual confounding cannot be excluded, and the observed associations should be interpreted cautiously.

In addition, the non-randomized selection of surgical approach introduces potential confounding by indication, as factors influencing the choice of operative technique may also have affected outcomes.

Although LOS represents a pragmatic measure of recovery, it should be interpreted as a surrogate outcome rather than a comprehensive measure of postoperative morbidity. In addition, important postoperative outcomes, including complications, readmission, reoperation, surgical-site infection, intra-abdominal abscess, ICU use, and mortality, were not consistently available and therefore were not assessed.

The definition of complicated appendicitis was restricted to abscess formation and generalized peritonitis, based on consistently documented intraoperative findings, and did not include other descriptors such as gangrene, phlegmon, appendiceal mass, or isolated perforation, which may limit comparability with studies using broader classifications.

Additionally, retrospective extraction from medical records may have introduced information bias or misclassification, particularly in the assessment of disease severity and variables dependent on operative documentation.

Furthermore, the study was conducted in a single center, which may limit the generalizability of the findings. Surgical procedures were performed by multiple surgeons with varying levels of experience, which may have influenced operative outcomes and length of hospital stay. The absence of long-term follow-up precludes assessment of late complications and outcomes beyond hospitalization.

Finally, although statistical adjustments were performed, the observational design does not permit definitive causal inference.

## 5. Conclusions

In this retrospective cohort, laparoscopic appendectomy was associated with a significantly shorter hospital stay compared with the open approach. However, disease severity and patient age were the main determinants of postoperative outcomes and prolonged hospitalization. Although laparoscopy was associated with favorable outcomes overall, this association appeared attenuated in more severe disease. Importantly, the laparoscopic approach remained independently associated with reduced length of stay after adjustment for measured confounders.

These findings suggest that differences in outcomes may be influenced primarily by disease severity and patient-related factors rather than surgical approach alone. Careful patient selection and surgical expertise remain essential to optimize outcomes. Overall, this study highlights the combined impact of age and disease severity on postoperative outcomes while supporting an independent association between laparoscopy and shorter hospital stay.

## Figures and Tables

**Table 1:** Baseline characteristics of the study population (n = 311).

Variable	Value
Total patients, n	311
Age, mean ± SD (years)	43.28 ± 17.97
Age range (years)	18–86
Male sex, n (%)	182 (58.5%)
Female sex, n (%)	129 (41.5%)
Complicated appendicitis, n (%)	120 (38.6%)
Uncomplicated appendicitis, n (%)	191 (61.4%)
Length of stay, mean ± SD (days)	5.09 ± 2.94

**Table 2:** Distribution of clinical forms of acute appendicitis.

Clinical Form	n	Mean Age (Years)	LOS (Days)
Uncomplicated appendicitis	149	39.53	4.23
Appendicitis with abscess	107	45.18	5.29
Appendicitis with generalized peritonitis	55	49.76	7.05

Note: LOS > 7 days occurred in: 2.0% (unspecified), 13.1% (abscess) and 29.1% (peritonitis).

**Table 3:** Baseline characteristics and comparison between open and laparoscopic appendectomy.

Variable	Open (n = 205)	Laparoscopic (n = 106)	*p*-Value
**Age, mean ± SD (years)**	**45.6 ± 18.1**	**38.9 ± 16.4**	**0.003**
**Male sex, n (%)**	**111 (54.1%)**	**70 (66.0%)**	**0.055**
**Complicated appendicitis, n (%)**	**93 (45.4%)**	**28 (26.4%)**	**0.001**
**Uncomplicated appendicitis, n (%)**	**112 (54.6%)**	**78 (73.6%)**	**0.001**
**Length of stay, mean ± SD (days)**	**5.68 ± 3.11**	**4.43 ± 2.62**	**<0.001**
**Conversion to open, n (%)**	**—**	**10 (9.4%)**	**—**

**Table 4:** Logistic regression analysis for predictors of complicated appendicitis.

Variable	OR	95% CI	*p*-Value
Age (per +10 years)	1.38	1.20–1.58	2.28 × 10^−6^
Male sex	1.18	0.73–1.91	0.506

Note: Model performance AUC = 0.669.

**Table 5:** Multivariable logistic regression for prolonged hospital stay (>7 days).

Variable	OR	95% CI	*p*-Value
Age (per +10 years)	1.63	1.29–2.05	3.37 × 10^−5^
Complicated appendicitis	3.88	1.67–9.01	0.00169
Male sex	0.71	0.32–1.58	0.395

Note: Model performance AUC = 0.803.

**Table 6:** Length of stay according to disease severity.

Group	Age, Mean ± SD (Years)	LOS, Mean ± SD (Days)	*p*-Value
Uncomplicated appendicitis	39.53 ± 17.58	4.23 ± 2.52	<0.001
Complicated appendicitis	49.53 ± 16.85	6.10 ± 3.27	—

**Table 7:** Statistical comparison between complicated and uncomplicated appendicitis.

Comparison	Test	Statistic	*p*-Value
Age: complicated vs. uncomplicated	Welch t	−5.09	1 × 10^−6^
Age: complicated vs. uncomplicated	Mann–Whitney	7474	<0.001
LOS: complicated vs. uncomplicated	Welch t	−4.69	5 × 10^−6^
LOS: complicated vs. uncomplicated	Mann–Whitney	6780	<0.001
Sex vs. complication	Chi-square	0.289	0.591

**Table 8:** Multivariable linear regression for predictors of LOS.

Variable	β Coefficient	95% CI	*p*-Value
Age (per +10 years)	+0.42 days	0.25–0.59	<0.001
Complicated appendicitis	+1.31 days	0.78–1.84	<0.001
Laparoscopic approach	−1.07 days	−1.65 to −0.49	<0.001
Male sex	+0.18 days	−0.22 to 0.58	0.37

**Table 9:** ROC analysis for prediction of complicated appendicitis using age.

Parameter	Value
AUC	0.669
Optimal cut-off (years)	47
Sensitivity	0.68
Specificity	0.61
Youden index	0.29

**Table 10:** Interaction analysis for predictors of LOS.

Variable	β	*p*-Value
Laparoscopic approach	−0.98	0.002
Complicated appendicitis	+1.27	<0.001
Approach × Complicated	+0.22	0.41

**Table 11:** Outcomes according to age group.

Variable	<60 Years	≥60 Years	*p*-Value
Patients, n	—	—	—
Complicated appendicitis, %	32.4%	54.1%	<0.001
LOS, mean ± SD (days)	4.62 ± 2.41	7.08 ± 3.36	<0.001
Conversion rate, %	7.1%	14.8%	0.08

## Data Availability

The data presented in this study are available on request from the corresponding author. The data are not publicly available due to patient confidentiality.
